# Abortion from a Legal Point of View

**Published:** 1908-06-20

**Authors:** 


### Medico-Legal Points.

### ABORTION FROM A LEGAL POINT OF VIEW.

Abortion, or miscarriage, as a legal term, means expulsion of the contents of the womb of a pregnant woman at any period of gestation short of the full term. To cause abortion is unlawful, unless it is done in good faith for the purpose of saving the life of the mother.

Any pregnant woman who, with intent to procure her own miscarriage, unlawfully administers to herself any poison or other noxious thing, or unlawfully uses any instrument, or other means whatsoever, is guilty of felony. Whosoever, with the intent to procure the miscarriage of any woman, whether pregnant or not, unlawfully administers to, or causes to be taken by her, any poison or other noxious thing, or unlawfully uses any instrument or other means whatsoever, is guilty of felony (24 and 25 Vict. c. 100 s. 58). These offences are punishable by penal servitude for life or not less than three years, or by imprisonment for not more than two years with or without hard labour.

On an indictment for administering feverfew and other drugs to procure abortion, it appeared that the prisoner gave the woman, who was alleged to be with child by him, two powders, with directions to take one on each of two successive nights, and said that the effect would be to cause miscarriage. She took one of the powders, with the feverfew, which brought on violent sickness. The other powder was examined by a physician, and he could not discover any mineral substance in it; as far as he could judge from the taste, smell, and appearance, it was a mixture of savin and fenugreek, the latter being the larger ingredient. The fenugreek would scarcely produce any effect at all; savin, in that quantity, might produce a little disturbance in the stomach for the time, but would do no further injury. Feverfew is a herb very similar to camomile; it is a tonic in common use among the peasantry, and has nothing noxious in it. A mixture of the powder and a decoction of this herb would not alter the properties of either. The prisoner upon two or three subsequent occasions had brought the woman other medicines to take for the same purpose, some of which she had taken, but not the rest. Chief Justice Wilde held that the evidence was not sufficient to prove that the drugs administered came within the meaning of the words "poison or other noxious thing" (*R.* v. *Perry*). He also held that the after transactions were admissible as showing the intent with which the particular drugs referred to in the indictment were administered. As the prisoner administered the drugs with intent to procure a miscarriage, and as savin is unquestionably in its nature a noxious drug, the decision in this case seems open to question.

A thing to be "noxious" must be such as, in the quantity administered or taken, is capable of causing harm. Manual delivery is not necessary to constitute "administration," nor need the accused be present when the substance is actually taken. Actual reception into the system is not necessary to constitute the offence. Where an instrument is used which might be innocently employed, the intention of the accused may be proved by evidence of his causing other miscarriages by the same means.

Upon an indictment, under 24 and 25 Vict. c. 100, s. 59, for applying a certain noxious thing, knowing that the same is intended to be used with intent to procure miscarriage, it is necessary to prove that the thing supplied is noxious. The supplying an innoxious drug, whatever may be the intent of the person supplying it, is not an offence against that enactment (*R.* v. *Isaacs*).

A man and a woman were jointly indicted for feloniously administering to C. a noxious thing to the jurors unknown, with intent to procure miscarriage. C., being pregnant, went to the male prisoner, who said he would give her some stuff to put her right, and gave her a light-coloured medicine, and told her to take doses of it till she became in pain. She did so, and it made her ill. She then went to him again, and he said the safest course would be to get her a place to go to. He told her that he had found a place for her at L., and gave her some more of the stuff, which he said would take effect when she got there. They went together to L., and met the female prisoner. C. then began to feel pain and told the female prisoner of it, whereupon the man told the latter what he had given C. They all went home to the female prisoner's, and the male prisoner then gave C. another bottle of similar stuff in the female prisoner's presence, and told her to take it like the other. She did so, and became very ill, and next day had a miscarriage, the female prisoner attending on her. It was held that there was evidence that the stuff administered was a noxious thing within sect. 58 (*R.* v. *Hollis*).

Where the prisoner caused half-an-ounce of oil of juniper to be administered, and it was proved that quantities considerably less may be taken without any ill-effect, but that half-an-ounce produces ill effects and is dangerous to a pregnant woman, it was held that there was evidence of the administering of a "noxious thing" within the section.

In order to bring a case within the 24 and 25 Vict. c. 100, s. 59, it is not necessary that the intention of using the noxious substance should exist in the mind of any other person than the person supplying it. A prisoner was indicted for supplying savin, knowing that it was intended to be unlawfully used to procure a miscarriage, and it was contended that there was no case against him, because it was necessary that he should know that the savin was intended to be used with intent to procure the miscarriage, whereas it was not intended, except by the prisoner himself, to be so used; the jury found that the case was in other respects proved, but that the prosecutrix did not intend to take the savin, nor did any other person except the prisoner, intend that she should take it, but upon a case reserved, it was held that the intent of any other person than the prisoner is not necessary to the commission of the offence. The statute is directed against the supplying of any substance with the intention that it shall be employed in procuring abortion. The prisoner, in this case, supplied the substance, and intended that it should be employed to procure abortion. He knew of his own intention, and was, therefore, within the words of the statute (*R.* v. *Hillman*).

The thing supplied with intent to procure abortion must be noxious in its nature. Where, therefore, an indictment charged the prisoner with supplying a certain noxious thing with intent to procure abortion, and a surgeon proved that the liquid was some vegetable decoction of a harmless character, and such as would not procure a miscarriage; but if taken with the belief that it would produce it, it might, by acting on the imagination, produce that effect; it was held that this liquid was not within the clause, although the woman proved that, after taking a wineglassful, she felt dizzy in the head when she went to bed, and felt stupid in the head the next morning.

To constitute an administering, or causing to be taken, it is not necessary that there should be a delivery by the hand. Where, therefore, on an indictment for administering poison, and causing poison to be taken, it appeared that the prisoner had mixed poison with coffee, and had told her mistress that the coffee was for her, and the mistress took it, and drank some of it; it was held that this was sufficient. A mere delivery to the woman, however, is not sufficient; the poison must be taken into the mouth, and, it seems, some of it swallowed, to constitute an administering (*R.* v. *Harley; R.* v. *Cadman*).

Upon an indictment for administering poison to Emma Chaney with intent to procure miscarriage, it appeared that she, being, and believing herself to be, pregnant, applied to the prisoner to get her something to procure her miscarriage, and that the prisoner accordingly purchased some preparation of mercury, which he gave to her, directing her to take one half of the quantity in gin; Chaney accordingly procured the gin, and in the absence of the prisoner, took the dose, which produced a miscarriage. The jury found these facts, and that the mercury was both given by the prisoner to Chaney, and taken by her, with intent to procure miscarriage. It was held that the prisoner was properly convicted (*R.* v. *Wilson*).

On an indictment for administering savin with intent to procure abortion, the administration of savin on one day was proved, and it was proposed on the part of the prosecution to prove the administration of similar drugs on many subsequent days, for the purpose of showing the intent, and also as part of the same felony, and it was urged that the substance of the felony was the administration of drugs for the purpose of procuring abortion, and if that were done by homœopathic doses, taken for a long period, all would form part of one felony; but Mr. Justice Cresswell held that other matters of the same description might be proved for the purpose of showing the intent, but that the administration of other savin on other days could not be given in evidence as part of the offence (*R.* v. *Calder*).

